# The Physician’s All-Requisite Time and Labor-Saving Account Book

**Published:** 1891-01

**Authors:** 


The Physician's All-Requisite Time and Labor-Saving Account-Book. Designed by William A. Siebert, M. D., Easton, Pa. Philadelphia and London: F. A. Davis, publisher.

Many books of this nature have been offered to the profession of late, but each one has some defect that prevents its universal adoption. After a careful examination of the one before us, we are quite of the opinion that it is one of the best, and comes nearer to the ideal than any book designed for a similar purpose that we have seen. This book has a great advantage in that it will last many years, its space is so condensed and utilized. It is issued in two sizes — 300 pages contain spaces for 900 accounts, price, $5; and 600 pages provide for 1,800 accounts; price, $8. The book must be examined to be appreciated, and is almost sure to command a purchaser wherever presented for inspection.

